# miRNA let-7e Modulates the Wnt Pathway and Early Nephrogenic Markers in Mouse Embryonic Stem Cell Differentiation

**DOI:** 10.1371/journal.pone.0060937

**Published:** 2013-04-10

**Authors:** Jose Luis Viñas, Marina Ventayol, Bernhard Brüne, Michaela Jung, Anna Sola, Felip Pi, Chrysoula Mastora, Georgina Hotter

**Affiliations:** 1 Departament of Experimental Pathology, Instituto de Investigaciones Biomédicas de Barcelona (IIBB-CSIC, IDIBAPS), Barcelona, Spain; 2 CIBER-BBN, Networking Center on Bioengineering, Biomaterials and Nanomedicine, Zaragoza, Spain; 3 Departamento de Fisiología, Universidad de Barcelona, Barcelona, Spain; 4 Institute of Biochemistry I/ZAFES, Goethe-University Frankfurt, Theodor-Stern-Kai, Frankfurt, Germany; University of Ulm, Germany

## Abstract

This study indicates that embryonic stem cells [ESCs] cultured with retinoic acid and activin A significantly upregulate the miRNA let-7e. This specific miRNA modulates the Wnt pathway and the expression of early nephrogenic markers under these differentiation conditions. The differentiation markers WT1, Pax2 and Wnt4 were downregulated when miRNA let-7e was silenced, thus indicating the role of miRNA let-7e in the differentiation process. PKCβ, GSK3β phosphorylation (GSK3β^P^) and β-catenin expression was reduced in differentiated cells and reversed by miRNA let-7e silencing. Addition of a PKCβ inhibitor to the miRNA let-7e silenced cells abolished let-7e-derived effects in differentiation markers, and reversed the increase in GSK3β^P^ and β-catenin, thus indicating that miRNA let-7e is involved in differentiation via the modulation of GSK3β phosphorylation and β-catenin production.

## Introduction

Mouse embryonic stem cells (mESCs) isolated from blastocysts are pluripotent cells with unlimited self-renewal capacity and the potential to generate all the cell types of the three germinal layers. These cells have been used to generate highly specialized cells and tissues *in vitro* such as pancreatic cells [1], motor neurons [2], hematopoietic cells [3] and renal cells [4].

Nowadays much research is trying to develop renal precursors that could integrate and regenerate damaged kidney. From our point of view it is important to study the possible mechanisms involved in ESCs differentiation because these cells could be a potential source of these precursors.

mESCs in cell culture remain undifferentiated in the presence of leukemia inhibitory factor (LIF) [5]. Withdrawal of LIF, gives rise to embryoid bodies (EBs) formation that can be differentiated toward renal lineage using activin A, retinoic acid and BMP7 [6]. Retinoic acid and activin A stimulate expression of early intermediate mesoderm markers on the basis of pioneering work in *Xenopus* embryos [7], [8] and in murine embryonic stem cells in a more recent study [9].

Stem cell differentiation towards renal lineage is associated with the sequential expression of different marker genes characteristic of early kidney development.

Pax2 is one of the earliest markers expressed in the intermediate mesoderm from where takes place the formation of the kidney. Pax2 and Wt1 are subsequently expressed in the metanephric mesenchyme and are two genes characteristic for initiation of nephrogenesis [9]. This gene expression is then followed by the secretion of many additional secreted factors, including Wnt4 and Wnt9b which are expressed in the condensing mesenchyme [10], [11] and both are involved in the formation of epithelia [11]. Following that, the presence of Notch2 directs cells primarily to the proximal tubule fate [11].

Wnt/β-catenin signalling is essential during kidney development as well as in cell differentiation towards renal lineage [12], [13]. Furthermore, Wnt is also believed to stimulate ESCs proliferation and maintain pluripotency [14], and its improper regulation is associated with cyst formation in the kidney [15]. Wnt/β-catenin activation should therefore be tightly regulated.

β-catenin production is dependent on Glycogen synthase kinase 3 beta (GSK3β) phosphorylation. GSK3β is a ubiquitously expressed, highly conserved serine/threonine protein kinase found in all eukaryotes and serves as a downstream regulatory switch for the Wnt signalling pathway [16]. Serine Phosphorylation of GSK3β is performed by protein kinase C beta (PKCβ) [17], [18].

microRNAs (miRNAs) are short noncoding RNAs of ∼22 nt that post-transcriptionally regulate gene expression through the 3′untranslated regions (3′UTRs) of their target mRNAs. miRNAs are able to regulate the expression of numerous mRNAs, some of them belonging to critical pathways during differentiation such as the Wnt Pathway [19]. Some of these miRNAs, as is the case of the miRNA let-7 family, regulate cell proliferation and differentiation during development in different species [20]. Specifically, let-7e was detected in the adult kidney [21] and recent studies have started to investigate its role in renal cancer [a state of cell dedifferentiation], outlining that let-7e is downregulated and associated with metastasis and poor prognosis [22].

We hypothesized that miRNA let-7e was determinant in stem cell differentiation and expression of early nephrogenic markers.Therefore, EBs were differentiated using retinoic acid and activin A, a classical combination that promote the expression of genes characteristic of the intermediate mesoderm. miRNA let-7e silencing decreased the expression of these differentiation markers. In addition, since PKCβ is an inductor of GSK3β phosphorylation (GSK3β^P)^, we hypothesized that miRNA let-7e could inhibit the formation of PKCβ protein that in turn decreases serine phosphorylation and the negative regulation of GSK3β activity, destabilizing β-catenin during the differentiation process in mESCs.

Here we present our findings concerning the involvement of miRNA let-7e in stem cell differentiation via the modulation of GSK3β phosphorylation and β-catenin production.

## Materials and Methods

### Ethics Statement

This study has been approved by the bioethics committee of CSIC (Spanish National Research Council).

### Culture of the Undifferentiated mESCs

All chemical reagents were obtained from Millipore unless otherwise indicated. mESCs (C57BL/6) were grown in DMEM (EmbryoMax® ES Cell Qualified DMEM) supplemented with 15% FBS, 1% non-essential nucleotides, 1% non-essential amino acids, 0.1 mM 2-Mercaptoetanol, 2 mM L-Glutamine, 100 units/ml Penicillin 100 µg/ml Streptomycin and 10^3^ units/ml mouse LIF (mLIF) on a 0.1% gelatine-coated tissue culture plate with a feeder layer of Premier mouse embryonic fibroblasts (PMEF-H). mESCs were dissociated with 0.05% trypsin-EDTA and transferred to a Petri dish to induce embryoid body (EB) formation.

### Induction of EBs Differentiation

EBs suspension was transferred to culture flasks coated with 0.1% gelatine and cultured for an additional 1 to 10 days with the described medium without mLIF and with presence or absence of: retinoic acid (ATRA) (Sigma-Aldrich) 0.1 µM and activin A Sigma-Aldrich)10 ng/ml.

### Transfection

Transfection of EBs with 62.5 nM LNA-antiLet-7e oligonucleotide: 5′–ACTATACAACCTCCTACCTC-3′ (Exiqon) or the respective control antimiR 5′-AGAGCTCCCTTCAATCCAAA-3′ (Exiqon) were carried out using Lipofectamine™ 2000 (Invitrogen), according to the manufacturer’s protocol. Medium was replaced after 6 h of transfection.

### Other Treatments

Protein kinase C inhibitor Gö 6983 (Sigma-Aldrich), inhibits PKCβ isozyme and was administered (7 nM) at the same time as the LNA-antiLet-7e (6 h).

6-bromoindirubin-3'-oxime (BIO), the specific pharmacological inhibitor of glycogen synthase kinase-3 (GSK-3) was administered (5 µM) for 24 h.

### Experimental Groups

EBs were cultured for 1 to 10 days under different conditions:

- I) **CONTROL.-** EBs treated with control antimiR
- II) **DIF**.- EBs cultured with addition of 0.1 µM retinoic acid (ATRA) and 10 ng/ml activin A and treated with control antimiR.
- III) **DIF- Let-7e**.- Same as group II but let-7e silenced with LNA-antiLet-7e.
- IV) **DIF- Let-7e+PKCβi**.- Same as group III but treated with the protein kinase C inhibitor Gö 6983 (7 nM) for 6 h.
- V) **DIF+BIO**.- Same as group II but treated plus 5 µM BIO (inhibitor of GSK3) for 24 h (from day 9 to day 10).

### RNA Extraction Reverse Transcription mRNA and RT-PCR

Total RNA was extracted using TRIZOL reagent (Invitrogen) and 1 µg was reverse transcribed using an iScript™ cDNA synthesis kit (Bio-Rad). Quantitative reverse transcription PCRs were performed using the TaqMan Universal PCR Master Mix (Applied Biosystems) and primers previously validated and provided by Applied Biosystems: Pax2 (Mm01217939_m1), WT1 (Mm00460570_m1), Wnt9b **(**Mm00457102_m1), Wnt4 **(**Mm01194003_m1) and Notch2 (Mm00803077_m1). Results were normalized to glyceraldehyde 3-phosphate dehydrogenase (GAPDH) (Mm99999915_g1).

### miRNA Microarray

A miRNA PCR Array System (SA Biosciences) that included specific primers for 88 different mouse miRNAs was performed according the manufacturer’s instructions. RNA extracted from a pool of four control EBs (Control EBs d8) and a pool of 4 differentiated EBs (DIF EBs d8) were profiled. The resulting data was normalized to the average of the four small nuclear housekeeping RNAs (snoRNA251, snoRNA202, snoRNA142 and U6 snRNA). Results have been deposited in the Array Express database, accession number (E-MTAB-1468). In order to validate the expression levels obtained in the microarray experiments, we used quantitative real-time RT-PCR, and Northern Blot analysis.

### Northern Blot

miRNA Northern Blot Assay Kit (Signosis) was performed using 5 µg total RNA according to the manufacturer’s protocol using a complementary probe to miRNA mmu-let-7e.

### Reverse Transcription and RT-PCR microRNA

Total RNA was extracted as described previously. Specific reverse transcription for miRNA mmu-let-7e and U6 small nuclear RNA (U6 snRNA) was performed using TaqMan® microRNA Assay (Applied Biosystems). The RT-PCR was performed using the TaqMan® Universal PCR Master Mix (Applied Biosystems) and results were normalized to U6 snRNA.

### Western Blot

EBs were lysed in buffer 7 M Urea, 2 M Tiourea, 4% [w/v] CHAPS, 40 mM Tris-base, pH 7.4, containing 1 mM PMSF, 10 mM DTT, 0.1% protease inhibitor cocktail (Sigma-Aldrich) and 1% phosphatase inhibitor cocktail 3 (Sigma-Aldrich) at 4°C. Measurement of protein concentration was done using Bradford [Bio-Rad]. 100 µg of proteins were electrophoresed on SDS-10% polyacrylamide gel and transferred to nitrocellulose membranes which were subsequently blocked with 5% BSA in 0.06% Tween-TRIS-buffered saline for 1 h at RT (or at 4°C for GSK3β^P)^. Membranes were incubated overnight at 4°C with the following primary antibodies: Phospho-GSK3β-S (1∶500) (Abgent), PKCβI (1∶500) (Santa Cruz), Anti-β-catenin (1∶400) (BD Bioscience), **Pax2 (1∶350) (Santa Cruz), Wt1 (1∶250) (Santa Cruz)** washed with TTBS, and incubated for 1 h at RT with horseradish peroxidase-conjugated anti-rabbit IgG (1∶2.000) (Sigma-Aldrich) for GSK3β^P^, PKCβI and WT1 with anti-mouse IgG (1∶2.000) (Sigma-Aldrich) for β-catenin or with anti-goat IgG (1∶2.500) (Sigma-Aldrich) for Pax2, followed by ECL-detection. Equal protein loading was verified by immunoblotting for β-actin (Sigma-Aldrich).

### Statistical Analysis

Data are expressed as means ± SE. Means of different groups were compared using one way ANOVA. The Student-Newman-Keuls test was used for the evaluation of significant differences between groups. The existence of significant differences was already assumed when P<0.05.

## Results

### ATRA and Activin A Treatment Differentiates EBs and Promote the Expression of Early Nephrogenic Markers and miRNA let-7e

**Figure 1:**
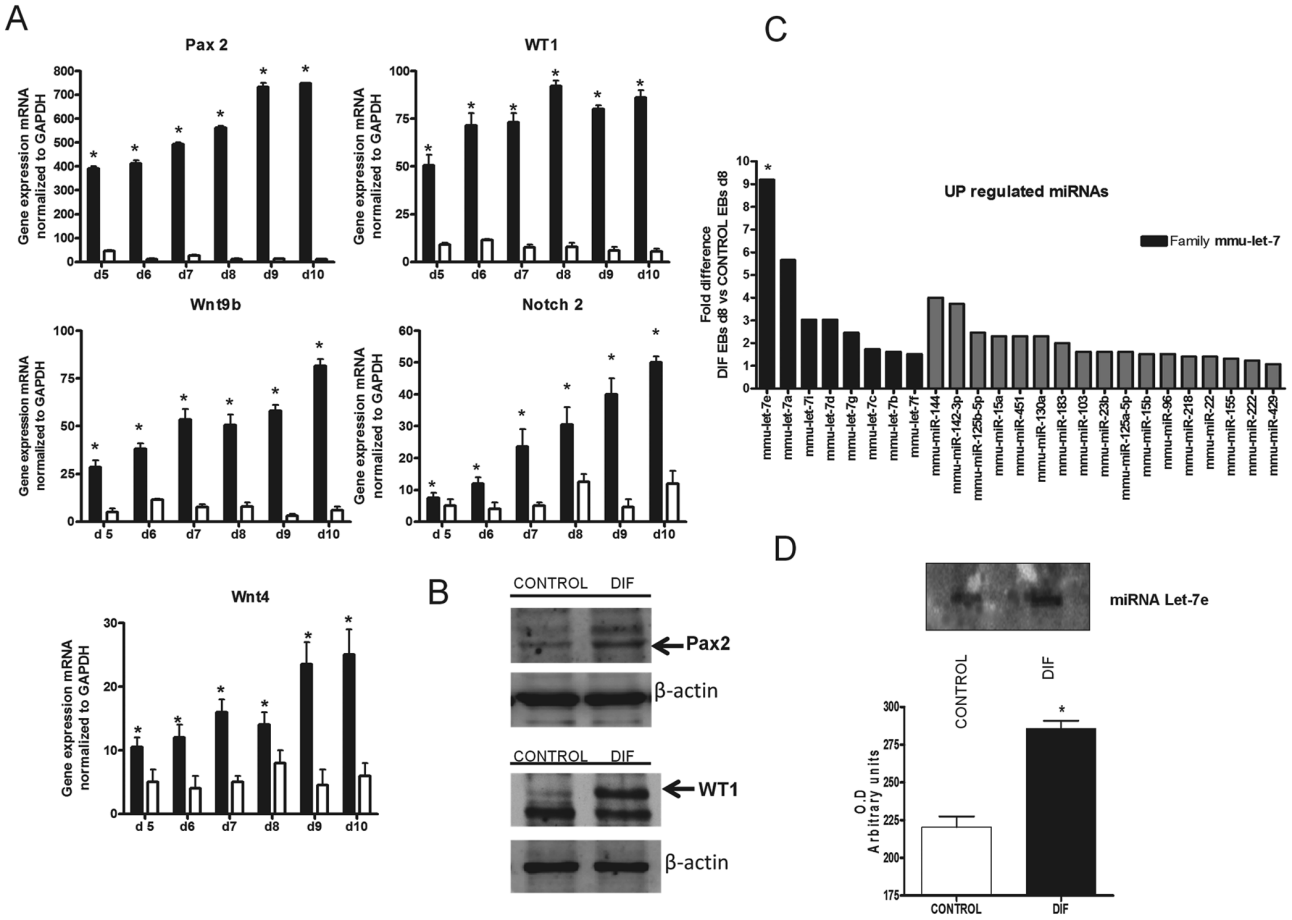
Embryoid bodies (EBs) cultured with a combination of ATRA and activin A express markers of intermediate mesoderm and metanephric mesenchyme and overexpresses the miRNA let-7e. **A)** Total RNA was extracted from EBs cultured for 1 to 10 days with ATRA and activin A [black columns] or without factors [white columns] and assayed for gene specific expression (Pax2, WT-1, Wnt4, Wnt9b and Notch2) by RT-PCR. Data shown starts on day 5, the time when expression of genes tested starts to be significantly upregulated. n = 4. Data are means ±S.D [*] P<0.05 vs. non treated EBs (white columns) and normalized to GAPDH; **B)** Western blot analysis of Pax2 and WT1 in cell lysates of EBs cultured for 8 days. Upregulated expressions of both proteins were detected in differentiated EBs. Western blots of β-actin as loading control are shown. **C)** miRNAs microarray in EBs cultured for 8 days with ATRA and activin A (DIF EBs) shows a positive fold difference >1.5 for all the family members of let-7 miRNAs tested (black columns), and in particular a (>9) fold difference for the miRNA let-7e (*) versus EBs cultured for 8 days without factors (CONTROL EBs). 88 different miRNAs were analyzed, but only the upreglated ones are shown. miRNA expression was normalized to the average of the four small nuclear housekeeping RNA (snoRNA251, snoRNA202, snoRNA142 and U6 snRNA). The fold difference for each miRNA was calculated using ΔΔCt method [ΔΔCt = ΔCt [DIF EBs] – ΔCt [CONTROL EBs]. **D)** Northern Blot probed for the mature miRNA let-7e in EBs cultured for 8 days with ATRA and activin A (DIF) or without factors (CONTROL) confirmed the result of the microarray. Result is graphed in Arbitrary units of optical density (O.D) n = 3±S.D [*] P<0.05 vs. CONTROL.

Figure 1A shows the efficiency of the treatment applied to embryoid bodies (EBs) to express intermediate mesoderm and methanephric mesenchyme markers. mRNA expression of Pax2 (intermediate mesoderm) and Pax2, WT1, Wnt9b, Wnt4, Notch2 [metanephric mesenchyme] increased significantly from day 5 to day 10 in differentiated EBs compared with non treated EBs (Fig. 1A). Protein expression of Pax2 and Wt1 also increased in differentiated EBs compared with non treated EBs (Fig. 1B).

A microRNA microarray was performed on EBs cultured for 8 days under differentiation conditions (he 8th day was selected based on the high expression of markers found that day). As shown in table 1, a total of 88 miRNAs were profiled, and a set of 26 miRNA were found upregulated. The expression levels of the miRNA let-7 family members were significantly upregulated; [>1.5] fold difference for the eight members (let-7e,let-7i,let-7a,let-7d,let-7g,let-7c,let-7b,let-7f) in treated EBs with respect the non treated EBs (Fig. 1C, Table 1). The most upregulated miRNA with a [>9] fold difference was the miRNA let-7e.

**Table 1:** Fold Up or Down difference expression of the 88 Microarray miRNAs.

miRNA ID	Fold Up-Regulation	miRNA ID	Fold Down-Regulation	miRNA ID	Fold Down-Regulation
	DIF EBs d8/CONTROL EBs d8		DIF EBs d8/CONTROL EBs d8		DIF EBs d8/CONTROL EBs d8
mmu-miR-292-3p	14263.1	mmu-miR-18a	−1	mmu-miR-128a	−2.83
mmu-let-7e	9.19	mmu-miR-9	−1	mmu-miR-185	−2.83
mmu-let-7a	5.66	mmu-miR-195	−1	mmu-miR-146b	−2.83
mmu-miR-144	4	mmu-miR-214	−1	mmu-miR-132	−3.03
mmu-miR-142-3p	3.73	mmu-miR-21	−1	mmu-miR-106a	−3.03
mmu-let-7i	3.03	mmu-miR-7a	−1.07	mmu-miR-208	−3.25
mmu-let-7d	3.03	mmu-miR-26b	−1.07	mmu-miR-322	−3.73
mmu-miR-125b-5p	2.46	mmu-miR-142-5p	−1.07	mmu-miR-503	−3.73
mmu-let-7g	2.46	mmu-miR-92a	−1.07	mmu-miR-10b	−4.92
mmu-miR-15a	2.3	mmu-miR-24	−1.07	mmu-miR-378	−5.28
mmu-miR-451	2.3	mmu-miR-146a	−1.15	mmu-miR-205	−5.66
mmu-miR-130a	2.3	mmu-miR-10a	−1.15	mmu-miR-20b	−6.96
mmu-miR-183	2	mmu-miR-16	−1.15	mmu-miR-182	−8.57
mmu-let-7c	1.74	mmu-miR-150	−1.23	mmu-miR-138	−8.57
mmu-miR-103	1.62	mmu-miR-1	−1.23	mmu-miR-375	−8.57
mmu-miR-23b	1.62	mmu-miR-140	−1.23	mmu-miR-101b	−9.19
mmu-miR-125a-5p	1.62	mmu-miR-301a	−1.23	mmu-miR-320	−9.19
mmu-let-7b	1.62	mmu-miR-17	−1.23	mmu-miR-370	−12.13
mmu-let-7f	1.52	mmu-miR-126-3p	−1.32	mmu-miR-488	−16
mmu-miR-15b	1.52	mmu-miR-106b	−1.41	mmu-miR-345-5p	−17.15
mmu-miR-96	1.52	mmu-miR-141	−1.41	mmu-miR-541	−18.38
mmu-miR-218	1.41	mmu-miR-219	−1.32	mmu-miR-122	−42.22
mmu-miR-22	1.41	mmu-miR-99a	−1.41	mmu-miR-133b	−48.5
mmu-miR-155	1.32	mmu-miR-203	−1.41	mmu-miR-33	−59.71
mmu-miR-222	1.23	mmu-miR-93	−1.52	mmu-miR-124	−97.01
mmu-miR-429	1.07	mmu-miR-192	−1.52	mmu-miR-129-5p	−111.43
		mmu-miR-100	−1.74	mmu-miR-210	−157.59
		mmu-miR-20a	−2	mmu-miR-134	−194.01
		mmu-miR-137	−2	mmu-miR-215	−222.86
		mmu-miR-194	−2.14	mmu-miR-452	−675.59
		mmu-miR-196a	−2.64	mmu-miR-223	−955.43

Differentiated sample versus control sample [DIF EBs d8/CONTROL EBs d8].

To confirm the results of the microRNA microarray, we examined expression levels of let-7e miRNA in treated and non treated EBs by northern blot analysis and additionally by real-time PCR. As presented in Fig. 1D, miRNA let-7e expression in treated EBs was dramatically upregulated on day 8 relative to non treated EBs.

### miRNA let-7e Overexpression is Involved in EBs Expression of Early Nephrogenic Markers

**Figure 2:**
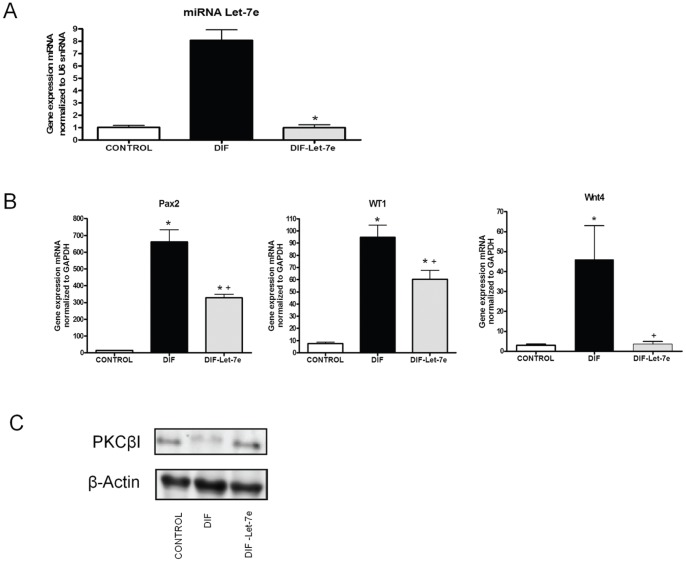
miRNA let-7e silencing reduces the expression of the differentiation markers WT1, Pax2, Wnt4 and increases the protein levels of PKCβ. Differentiated EBs were collected on day 10 and LNA-antiLet-7e was added on days 7 and 9 of differentiation to collect the cells after 24 h of the last transfection (day 10). Groups: Non treated EBs (CONTROL), differentiated EBs+control antimiR (DIF), differentiated EBs and miRNA let-7e silenced (DIF-Let-7e). **A)** RT-PCR of the mature miRNA let-7e confirms that miRNA let-7e is silenced after the administration of antiLet-7e. n = 3. Data are means ±S.D (*) P<0.05 vs. DIF and normalized to U6 snRNA; **B)** RT-PCR of WT1, Pax2 and Wnt4 show that miRNA let-7e silencing provokes a downregulation of these genes. Levels were normalized to GAPDH and data represent the mean of three independent experiments ±S.D (*) P<0.05 versus CONTROL, ±S.D (+) P<0.05 versus DIF. **C)** PKCβ levels evaluated by Western blot analysis show a downregulated expression in differentiated EBs (DIF) and a recovery of the expression in silenced EBs (DIF-Let-7e). Upper panel: Western blot analysis of PKCβ. Lower panel: Western blot analysis of β-actin.

In order to evaluate the role of miRNA let-7e during the EBs differentiation, let-7e was silenced. Efficient silencing of miRNA let-7e was observed since expression levels of miRNA let-7e decreased significantly after silencing (Fig. 2A).

When miRNA let-7e was silenced, mRNA expression of the differentiation markers WT1, Pax2, Wnt4 were significantly decreased on day 10 (Fig. 2B), thus indicating the role of miRNA let-7e in EBs differentiation. Similar results were observed on day 6 (data not shown).

In addition miRNA let-7e is an upstream regulator of PKCβ since Western blot analysis of PKCβ, revealed a decrease in the expression in differentiated EBs and an increase when miRNA let-7e was silenced (Fig. 2C) indicating the inhibitory influence of miRNA let-7e on PKCβ levels.

### miRNA let-7e Inhibitory Effect on PKCβ Prevents Phosphorylation of GSK3β and β-catenin Formation

**Figure 3:**
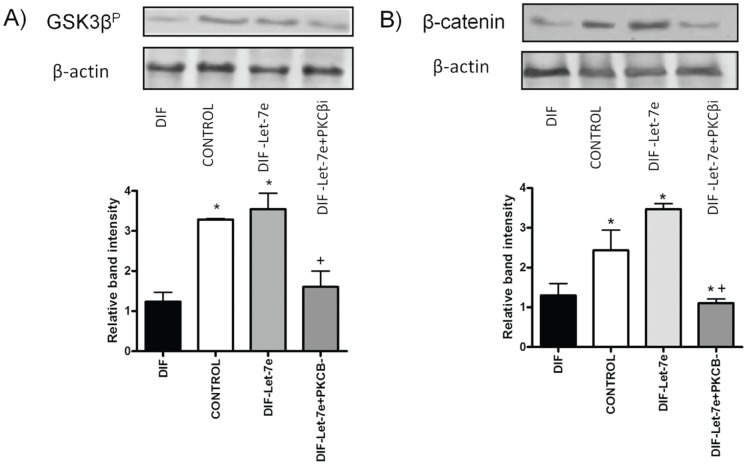
Western blot analysis of GSK3β serine phosphorylation (GSK3β^P^) (A) and β-catenin (B) in cell lysates of EBs cultured for 10 days. Downregulated expressions of both GSK3β^P^ and β-catenin were detected in differentiated EBs and the expressions were recovered when LNA-antiLet-7e (DIF-Let-7e) was added. Inhibition of PKCβ in the differentiated EBs (DIF-Let-7e+PKCβi) provokes a downregulation again. Western blots of β-actin as loading controls are shown. n = 3 (*) P<0.05 versus DIF, (+) P<0.05 versus versus DIF-Let7e. Groups: differentiated EBs+control antimiR (DIF), Non treated EBs (CONTROL), differentiated EBs+LNA-antiLet-7e (DIF-Let-7e), differentiated EBs+LNA-antiLet-7e and PKCβ inhibitor (Gö 6983) (DIF-Let-7e+PKCβi).

The differentiation treatment applied to embryoid bodies (EBs) provokes a decrease in GSK3β serine phosphorylation (GSK3β^P^])and β-catenin expression compared to EBs control. Conversely, miRNA let-7e silencing induces an increase in the expression of GSK3β^P^ and β-catenin, indicating the relation between miRNA let-7e and Wnt pathway (Fig. 3).

Addition of a PKCβ inhibitor to the miRNA let-7e silenced group reversed the increase in GSK3β^P^ and β-catenin, thus indicating the direct relationship between PKCβ, GSK3β^P^ and β-catenin levels.

**Figure 4:**
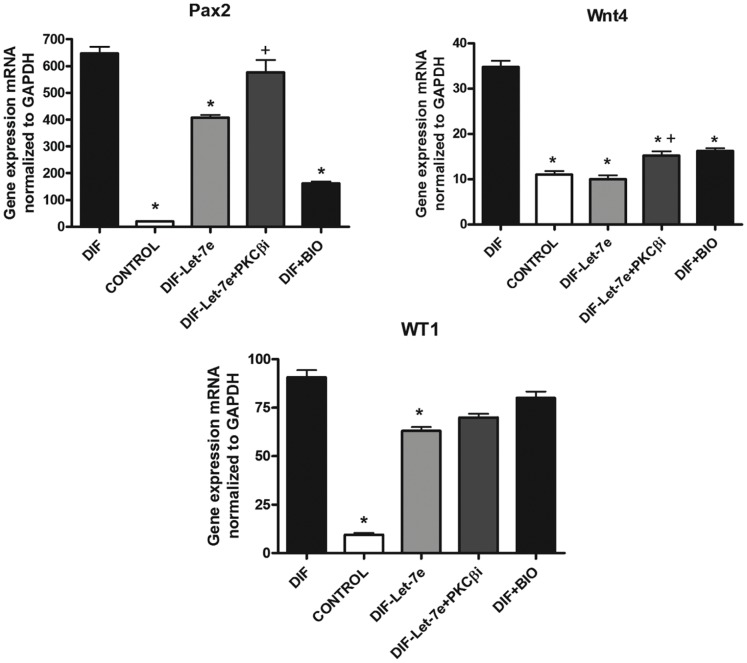
RT-PCR of Pax2, Wnt4 and WT1. miRNA let-7e silencing (DIF-Let-7e) and BIO (DIF+BIO) provokes a downregulation of the genes Pax2, Wnt4 and WT1. Inhibition of PKCβ (DIF-Let-7e+PKCβi) provokes a recovery in the expression of these genes. Levels were normalized to GAPDH and data represent the mean from three independent experiments ±S.D (*) P<0.05 versus DIF, (+) P<0.05 versus DIF-Let7e. Groups: differentiated EBs+control antimiR (DIF), Non treated EBs (CONTROL), differentiated EBs+LNA-antiLet-7e (DIF-Let-7e), differentiated EBs+LNA-antiLet-7e and PKCβ inhibitor (Gö 6983) (DIF-Let-7e+PKCβi), differentiated EBs+BIO (DIF+BIO).

Addition of a PKCβ inhibitor to the miRNA let-7e silenced group reversed the decrease in differentiation markers provoked previously by let-7e silencing (Fig. 4). The addition of the Wnt pathway activator BIO to differentiated EBs, decreased the expression of differentiation markers.

## Discussion

In this study we differentiate EBs towards cells that express markers of intermediate mesoderm and metanephric mesenchyme using retinoic acid and activin A [9]. After this treatment, we detected increases in the expression of some differentiation markers such as Pax2 and WT1, characteristics of the metanephric mesenchyme and related to the initiation of nephrogenesis [9].

Wnt9b and Wnt4, expressed in the condensing mesenchyme [10], [11], and Notch2, a gen involved in the proximal tubule fate determination [11] increased significantly during the differentiation protocol. Similar to our results, other authors, using an identical protocol, differentiated ESCs towards intermediate mesoderm-like cells. Furthermore, they obtained terminally differentiated renal cells with the subsequent addition of conditioned medium [9].

Our work indicates that activin A and retinoic acid provokes the differentiation towards cells that express markers of intermediate mesoderm and metanephric mesenchyme.

A microarray analysis revealed the overexpression of different miRNAs in the differentiated EBs compared to control EBs. Among the miRNAs overexpressed, we found let-7e, let-7i, let-7a, let-7d, let-7g, let-7c, let-7b and let-7f.

miRNA let-7 family was observed to regulate proliferation and differentiation of neural stem cells [23]. Here we show that let-7e is involved in the expression of early nephrogenic markers in mESCs, since let-7e miRNA silencing reduced the expression of Pax2, WT-1 and Wnt4, thus indicating the role of let-7e in differentiation.

PKCβ is a multifunctional serine/threonine protein kinase that plays important roles in the regulation of cell cycle, differentiation and proliferation [24]. Recently its inhibition has also been associated with T cell differentiation [25], osteogenic differentiation [26] or cardiomyocyte differentiation [27]. Moreover, the inhibition of PKCβ could block GSK3β serine phosphorylation, since it has been described that GSK3β phosphorylation by cholinergic stimulation is probably mediated by a mechanism involving protein kinase C [PKC], as it was blocked by a PKC inhibitor [17]. A close relationship between PKCβ protein expression and miRNA let-7e has been detected in our work, thus miRNA let-7e is an upstream regulator of the PKCβ protein expression. The fact that PKCβ has been listed in the microRNA.org database as one potential target gene of the miRNA let-7e reinforce our findings.

GSK3β constitutively phosphorylates the β-catenin protein and promotes its degradation. However, serine phosphorylation of GSK3β provokes the opposite effect and β-catenin degradation is avoided [28]. Our results show an increased expression of GSK3β serine phosphorylation and β-catenin, concomitant with a decrease in the expression of differentiation markers when let-7e is silenced. Therefore indicates that let-7e acts in differentiation as an inhibitor of β-catenin through the reduction of the inactive form of GSK3β.

The use of the PKCβ inhibitor Gö 6983 that acts avoiding phosphorylation of several PKC isoforms, including PKCβ [29], provoked a decrease in the Wnt pathway components, GSK3β serine phosphorylation and β-catenin, concomitant with an increase in the expression of the differentiation markers, indicating the relationship between PKCβ and Wnt pathway in this process.

Our results indicate that Wnt/β-catenin signalling is reduced in differentiated EBs. Moreover BIO, an activator of Wnt signaling pathway [30], reduced the expression of differentiation markers. In line with our work, it has been described that Wnt signalling activators such as BIO induced the maintenance of pluripotency in human and mouse embryonic stem cells [14], [28] and stimulates proliferation [31].

In the other hand, Wnt pathway is essential during kidney development and in cell differentiation toward renal lineage since it has been shown that *Wnt9b^−/−^* mice die within 24 h of birth due to agenesis of the kidneys [12] and also targeted disruption of *Wnt4* results in kidney agenesis and impairs mesenchymal-to-epithelial transition [32]. Thus it can be argued that activation of the Wnt pathway should be present in embryonic stem cell differentiation but in a way in which the end result of β-catenin production is well balanced in order to avoid pluripotency [31]. This work could well indicate that one potential interfering factor favoring the correct balance between Wnt signaling and β-catenin production is the miRNA let-7e.

In summary, our results indicate that miRNA let-7e is critically involved in the expression of early nephrogenic markers during EBs differentiation and the concomitant reduction of β-catenin production. The inhibitory effect of miRNA let-7e on GSK3β serine phosphorylation through PKCβ could explain these effects.
